# Genetic Basis and Molecular Mechanisms of Uveal Melanoma Metastasis: A Focus on Prognosis

**DOI:** 10.3389/fonc.2022.828112

**Published:** 2022-04-11

**Authors:** Carla Enrica Gallenga, Elena Franco, Ginevra Giovanna Adamo, Sara Silvia Violanti, Paolo Tassinari, Mauro Tognon, Paolo Perri

**Affiliations:** ^1^Department of Medical Sciences, University of Ferrara, Ferrara, Italy; ^2^Department of Translational Medicine and for Romagna, University of Ferrara, Ferrara, Italy; ^3^Department of Specialized Surgery, Section of Ophthalmology, Sant’Anna University Hospital, Ferrara, Italy; ^4^Department of Head and Neck, Section of Ophthalmology, San Paolo Hospital, Savona, Italy; ^5^Department of Neuroscience and Rehabilitation, Section of Ophthalmology, University of Ferrara, Ferrara, Italy

**Keywords:** uveal melanoma (UM), metastasis, molecular mechanism, prognostic markers, genetic analyses

## Abstract

Uveal melanoma (UM) is the most frequently found primary intraocular tumor, although it accounts for only 5% of all melanomas. Despite novel systemic therapies, patient survival has remained poor. Indeed, almost half of UM patients develop metastases from micro-metastases which were undetectable at diagnosis. Genetic analysis is crucial for metastatic risk prediction, as well as for patient management and follow-up. Several prognostic parameters have been explored, including tumor location, basal dimension and thickness, histopathologic cell type, vascular mimicry patterns, and infiltrating lymphocytes. Herein, the Authors review the available literature concerning cytogenetic prognostic markers and biochemical pathways correlated to UM metastasis development.

**Graphical Abstract:**
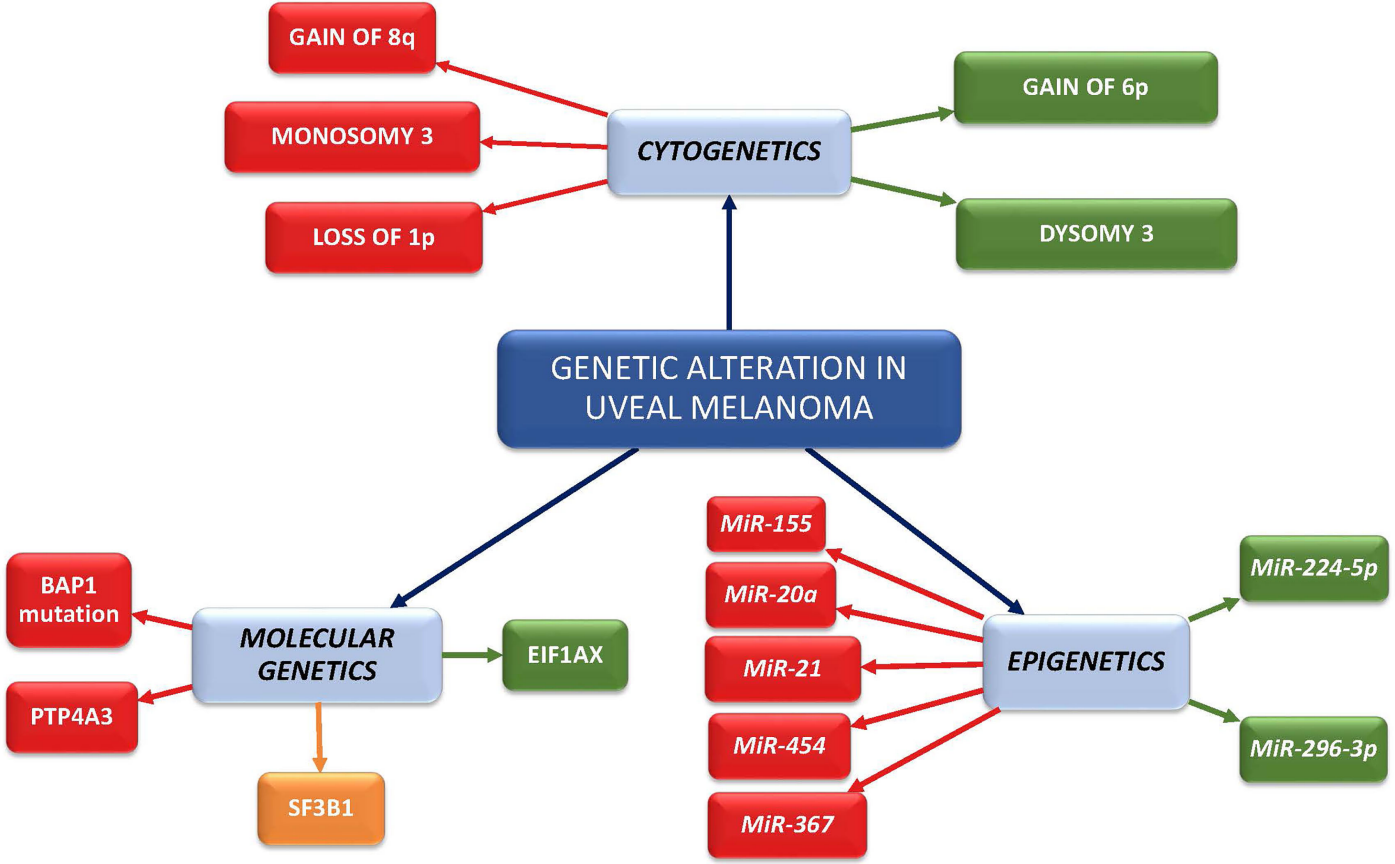
Red boxes represent high metastatic risk factors; orange box represents intermediate metastatic risk factor (late onset metastasis); green boxes represent low metastatic risk factors.

## 1 Introduction

Uveal melanoma (UM), though a relatively rare disease, is the most frequent primary malignant eye tumor in Caucasian adults. While local tumor control is outstanding, metastasis-related mortality remains relatively high. Almost 50% of patients develop metastases within 10 years of diagnosis, commonly in the liver (89%), lung (29%) and bone (17%) (1, 2) regardless of the type of treatment (3).

**Figure 1:**
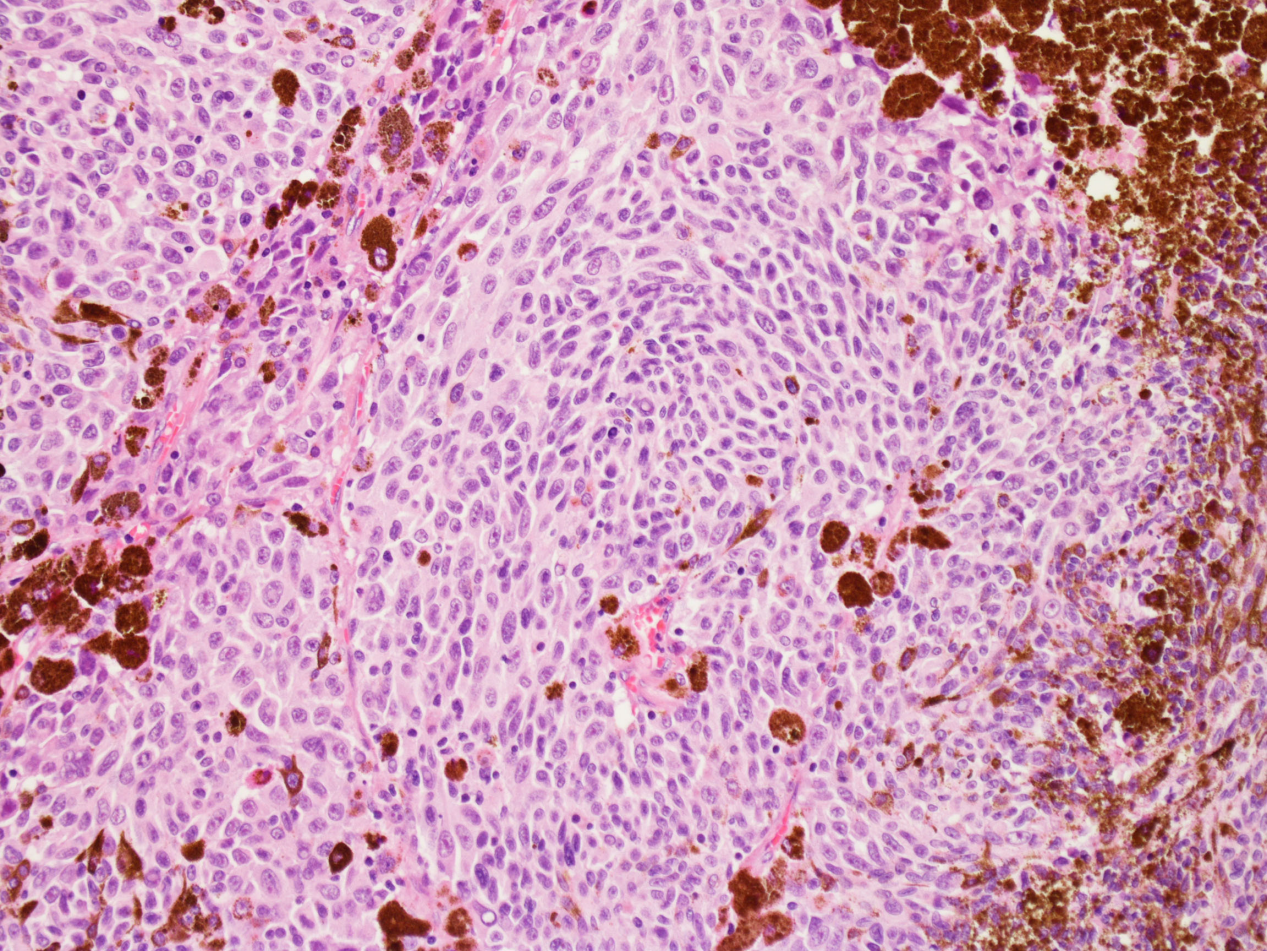
Haematoxylin-eosin histopathological analysis of uveal melanoma. (With permission of Prof. Giovanni Lanza, Institute of Pathological Anatomy, University of Ferrara).

**Figure 2:**
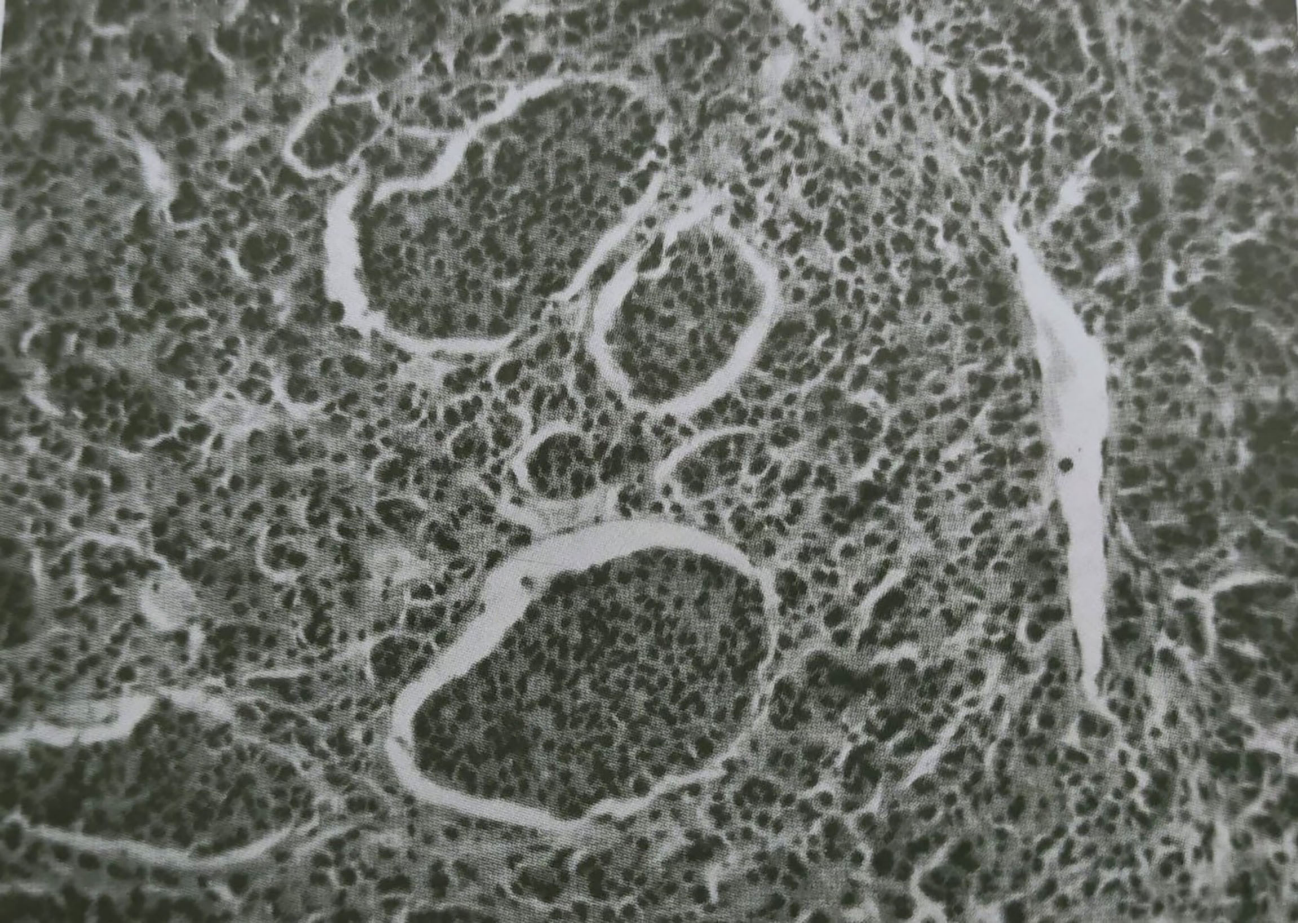
Malignant choroidal melanoma showing masses of neoplastic epithelioid cells invading a tumor vessel lumen. (Reproduced with permission from Prof. Gallenga P.E., Rass. Ital. Ottal.; published by Clinica oculistica dell’Università di Torino, 1961).

Several histopathological, clinical, radiological, cytogenetic and gene expression features can be used to estimate patient metastatic risk and prognosis (4) (**Figures 1**, **2**).

For example, cell type, tumor basal diameter, location of tumor epicenter, anterior tumor extension and tumor diameter, are known to correlate with distinct risk classes of melanoma-related survival (5, 6). Other features have also been studied, including Ki-67 proliferation index, inflammatory phenotype, deeper scleral extension, mixed/epithelioid cell type, as well as extracellular matrix patterns and high mitotic figures (7).

Additionally, certain clinical characteristics have been proven to be outcome predictors for UM patients: developing UM before the age of 21 is associated with better prognosis, compared to middle or older-age patients (8), while metastatic risk seems higher for males than females (9, 10).

Cytogenetic studies have highlighted the role of genetic factors, such as chromosomal aberrations and genetic mutations, in predicting patient survival. Indeed, tumors with monosomy 3 or gain of 8q are associated with poor prognosis. Monosomy 3 is also an independent risk factor for metastasis, if corrected for tumor site and diameter, whereas it correlates with significantly reduced disease-free survival (11). On the other hand, gain of 6p correlates with good prognosis, while loss of chromosome 1p and chromosome 8 have significant prognostic value, independently (12, 13). Single Nucleotide Polymorphism (SNP)-array analysis seems to be the most sensitive technique for investigating these chromosomal aberrations (14).

As for prognostic gene mutations, BAP1 loss (BRCA1-associated protein, located on chromosome 3), strictly correlates with metastasis development and poor prognosis. Additionally, BAP1 loss is associated with monosomy 3 (15–17).

Finally, UM patients can be classified based on gene expression profiling through high-resolution, genome-wide Comparative Genomic Hybridization (CGH) or SNP array techniques (18–20), and Multiplex ligation dependent probe amplification (MLPA) (21). Two profiles can be distinguished: class 1 tumors lead to good overall survival and low metastatic risk, whereas class 2 tumors are more likely to metastasize (20, 22). Alongside gene expression profiling, the ability to differentiate these two subgroups also relies on mutational status and micro-RNA expression analysis (23), proving that the deregulation of certain microRNAs has prognostic significance (24).

In this review, the genetic and molecular basis of uveal melanoma is reported on, with a focus on biomarkers that appear to underline prognosis and metastasis.

## 2 Pathological and Radiological Prognostic Factors

A multidisciplinary approach is mandatory for an accurate diagnostic and therapeutic management of UM.

Classical histologic findings have an impact on patient outcome, such as cell type, greatest thickness and largest basal diameter of the tumor, proliferative activity (both mitotic index and Ki67 proliferation rate) and specific growth pattern. Other useful histopathologic data are the deeper scleral extension, particularly the possible evidence of extraocular extension, with infiltration of the sclera along emissary nerves and vessels. Tumors confined to the choroid have basically a more favorable prognosis than melanomas extending into the ciliary body, so that the exact location of the lesion is important too (25).

The potential presence of prominent tumor-infiltrating lymphocytes and tumor-associated macrophages should be reported, as they seem to have a correlation with a higher risk of metastasis. A strong independent association with tumor-related mortality is linked to closed loops of PAS-positive material encircling tumor cells. Highly invasive tumor cells give rise to these loops through a mechanism of vasculogenic mimicry, allowing conduction of red blood cells and plasma cells (26, 27).

Measurements of UM are routinely acquired by US high frequency probes. Nevertheless, large tumors may have basal diameter too large for the limited field of view of US transducer and 2D US measurements of irregular shapes may be operator dependent. Accurate 3D volumetric data obtained through MRI are useful to evaluate the maximum tumor thickness (including sclera thickness), basal diameters of UM and possible extra-scleral or orbital extension (28, 29).

Some authors argue that a high degree of pigmentation (depending on the melanin content) may be associated with a less favorable prognosis. MRI can provide a quantitative evaluation of whole lesion pigmentation that seems to be more related to histopathologic examination. On the other hand, ophthalmoscopy allows a qualitative assessment of the melanin distribution, evaluating only the ventral portion of the tumor (30).

MRI should be included in the diagnostic management of selected UM. Thus, its role would be expanded from diagnostic to prognostic, with a positive impact on the therapeutic personalized planning (31).

## 3 Genomic Abnormalities in Uveal Melanoma

### 3.1 Cytogenetics

Cytogenetic rearrangements in UM have been extensively studied and mainly affect chromosomes 1, 3, 6, and 8. Among those studied, monosomy 3 appears as an early event in 50–60% of tumors and is often associated with isochromosome 8q. Isochromosome 8q is prone to segregate abnormally during mitosis and generates high levels of 8q gain. The correlation between these rearrangements and the risk of metastasis has been studied in the literature (11). Despite its pivotal role in the early identification of chromosome abnormalities in UM, karyotypic analysis has the serious limitation of being applicable only to large tumor samples obtained by enucleation and local resection (11).

Recently, several authors have focused their attention on the genetic evolution of uveal melanoma, which leads from primary tumor to metastatic disease. This is confirmed by the fact that metastasis show different mutations than primary tumors (32).

The evolutionary pathway starts with activation of the G-protein alfa signaling cascade, through a series of different harbor mutations that are mutually exclusive. After, there are further somatic mutations affecting BAP1, SF3B1 or EIFA1AX (see below), while cytogenetic rearrangements occur later, as an intermediate point of the evolutionary cascade and before the metastatic dissemination. It has to be noted that sometimes the latter can precede 8q gain (32).

Below we list the main cytogenetic alterations with the corresponding prognostic value.

#### 3.1.1 Monosomy 3

For almost 25 years, monosomy 3 has been known to strongly correlate with metastasis development (33) as well as clinicopathologic features indicating poor prognosis, for instance, ciliary body involvement, large diameter, high mitotic activity, extra-scleral extension, vascular loops, and epithelioid histology (34). On the other hand, metastases rarely develop in tumors with disomy 3 (13, 35).

Whether this abnormality in monosomy 3 tumors is homogeneously present has been a matter of debate (36). Patients with uveal melanoma carrying complete monosomy 3 determined by Fine Needle Aspiration Biopsy (FNAB) have substantially poorer 3-year prognosis than those with partial monosomy 3 or disomy 3. In particular, the cumulative probability for metastasis at 3 years is 2.6% for disomy 3, 5.3% for partial monosomy 3 (equivocal monosomy 3), and 24.0% for complete monosomy 3. Clinical outcomes for patients with partial monosomy 3 or disomy 3 do not significantly differ (37).

When there is a normal copy number of chromosome 3, tumors can show other chromosome alterations, such as 6p gain and 1p loss (11, 13).

#### 3.1.2 Isochromosome 8q

Chromosome 8 is also commonly altered in UM (38). Gain of the long arm of chromosome 8 (8q), which often results from isochromosome formation, occurs in 37% to 63% of primary UM (10, 11, 17, 39–42) and is associated with poor prognosis.

The frequent coexistence of 8q and monosomy 3 gain is associated with higher metastatic rates than a single aberration (18, 43): 5-year mortality rate is reported to be 66% in cases of concomitant monosomy 3 and 8q gain, 40% in cases of monosomy 3 and 31% in cases with 8q gain (34, 44).

In a recent study, Dogrusoz et al. investigated whether information on chromosome 3 and 8q status could enhance the prognostic value of the American Joint Committee on Cancer (AJCC) staging system. In the study cohort of 470 UMs with known chromosome 3 and 8q status, tumors with monosomy 3 and 8q gain showed an increased risk of metastatic death (40).

It has not yet been fully clarified which is the first chromosomal alteration in UM malignant transformation. Some authors have found that monosomy 3 is the first step and that 8q gain occurs subsequently (19), but others highlighted that 8q gain precedes chromosome 3 loss (45). Finally, other authors reported that the gain of the telomeric part of 8q is present in 92% of studied UM, so probably it has a central role in UM tumorigenesis (46).

#### 3.1.3 Loss of the short arm of chromosome 1

Loss of the short arm of chromosome 1 (1p) is frequently associated with monosomy 3 in 19–34% of UM and in 33% of metastasizing tumors (10, 11, 39, 41, 47). The concurrent loss of 1p and monosomy 3 has been reported to be an independent prognostic parameter for disease-free survival (11).

#### 3.1.4 Gain of 6p

Gain of the short arm of chromosome 6 (6p) was the first chromosome aberration to be reported in UM. The prevalence of this mutation is between 18% and 54% in UM and is associated with good prognosis (10, 11, 39, 41, 47).

Monosomy 3 or 6p gain are both very early events in tumorigenesis and they are probably involved in two mutually exclusive evolutionary pathways, as the occurrence of both of them is reported to be only in 4% of UM (18, 19, 48, 49).

### 3.2 Genome-Wide DNA Copy Number Profiling

#### 3.2.1 Genomic Classification of Tumors

Recently, FNABs from smaller tumors *in vivo* have been analyzed, using genome and expression profiling on microarrays, enabling precise analysis of combined chromosome imbalances in UM to be carried out (18–20, 50, 51). CGH, single nucleotide polymorphism (SNP) arrays and MLPA technique produce high-density, genome-wide DNA copy number profiles (21).

SNP array analyses have allowed the allelic status of chromosome 3 to be determined, showing that 3-5% of all UM exhibit isodisomy 3 (20, 52), in which one copy of chromosome 3 is lost, whereas the other copy is duplicated, with the same prognostic value as monosomy 3. This abnormality is copy-neutral, and therefore undetectable by CGH array or MLPA (38). Therefore, SNP array appears to be the most sensitive technique for investigating these prognostic correlations, thanks to its ability to detect chromosome 3 isodisomy.

Microsatellite analysis, MLPA, or high-resolution genome-wide techniques have shown the existence of partial deletions of chromosome 3, in either or both arms, but its prognostic implications are still under review (20, 51–55). A minimal region of deletion in 3p26.3 has also been found in liver metastases (20).

Onken et al. classified UMs in 2 groups based on gene expression profile: class 1 tumors (40%), with monosomy 3 and low metastatic risk, and class 2 tumors (60%), with 2 copies of chromosome 3 and high metastatic risk (50). Expression profiling showed that class 1 tumors were mainly characterized by a gain of 6p and 8q distal from band q21, while class 2 tumors revealed a gain of the entire 8q. It is worth noting that a minority of class 1 tumors, probably about 15%, is able to metastasize, as confirmed by class 1 profiles observed in some liver metastases (20).

Sometimes the small sample size obtained with FNABs can lead to wrong genetic categorization related to genetic heterogeneity of the tumor. Bagger et al. highlighted the importance of transvitreal retinochoroidal biopsy (TVRC) in obtaining larger and more representative tissue samples. The authors recommended a combination of Fluorescence in Situ Hybridization (FISH) and MLPA to better identify patients with a higher risk of developing metastasis and founded a genetic heterogeneity of chromosome 3 in a minority of tumors (56).

#### 3.2.2 Candidate Genes

Molecular genetics analyses with DNA sequencing techniques have identified several alterations that seem to play a role in the metastatic UM. The incidence of metastasis in UM is partly determined by random variables, related to mutation rate and type of mutation involved. It has been reported that smaller tumors have a higher mutation rate, while mutation type can affect the timing of onset of metastasis (57).

Van Raamsdonk was the first to describe the GNAQ/GNA11 mutations in UM (58). GNAQ and GNA11 are the driver mutations in 71-93% of UMs (59). They are mutually exclusive in the vast majority of tumors (7, 60–62), but it seems that they are not associated with metastasis and survival in UM (57).

Secondary driver may affect BAP1, SF3B1 and EIF1AX (~45%, ~25%, and ~20% of the primary posterior UM, respectively), which are generally mutually exclusive and have prognostic value (60). In particular, the Rotterdam Ocular Melanoma Study Group highlighted as association among BAP1 mutation and early metastasis with decreased survival rate; SF3B1 mutations with late-onset metastasis; and EIFAX mutations with low metastatic risk and longer disease-free survival (63).

P53 and BRCA pathways may contribute to unfavorable prognosis only in a subset of patients (64).

Additional mutations have been recently described: loss of heterozygosity over the GNAQ locus, loss-of-function mutations affecting CDKN2A*, *PBRM1*, *PIK3R2, and PTEN, and gain-of-function mutations affecting EZH2*, *PIK3CA, and* *MED12. The majority of them has been found in just one region of the primary tumor or private to their metastases; for this reason, they may be considered as tertiary driver mutations and are supposed to arise later during progression (32).

##### 3.2.2.1 GNAQ and GNA11

GNAQ and GNA11 code for the alpha-subunit of the heterotrimeric GTP-binding protein that couples G-protein-coupled receptor signaling to the RAS-RAF-MEK-ERK (MAPK) pathway. Alterations in this pathway are considered as an early event in cancer development and lead to the activation of multiple cascade pathways involved in cell growth and proliferation (7).

GNAQ mutation can be observed in all stages of malignant progression, suggesting it is an early event in UM, but uncorrelated with disease free survival (65). On the other hand, GNA11 mutations may occur at different stages of UM progression.

Most uveal nevi show either GNAQ or GNA11 mutations, while GNA11-mutated tumors might be more aggressive than GNAQ-mutated forms. This is probably related to the fact that GNAQ, differently from GNA11, requires a second hit to be fully activated (32). Despite these mutations do not seem to have meaningful prognostic value (66–68), recent data highlight the sensitivity of GNAQ and GNA11 mutations to MAP kinase, protein kinase C, AKT and YAP inhibitors. It is therefore not excluded that the analysis of GNAQ/GNA11 mutation could become a routine diagnostic-therapeutic test for UM (69).

##### 3.2.2.2 BAP1

BAP1 is a tumor-suppressor gene located on chromosome 3 (3p21.1), which encodes for a nuclear deubiquitinase involved in cell growth and cancer pathogenesis (17, 70). BAP1 mutation or inexpression are associated with high metastatic risk (71, 72). In up to 84% of metastasizing, class-2 UM, BAP1 inactivation coincides with the onset of metastatic behavior (16, 17). Furthermore, tumors presenting monosomy 3 and BAP1 mutations are characterized by decreased disease-free survival rates (73). No correlation between GNAQ and BAP1 has been reported (70, 74).

A germline mutation of BAP1 can be detected in rare cases. It correlates not only with a higher risk of UM, but also with other tumors. Under these circumstances it may be considered a broader approach, comprising genetic counselling and screening of family members (75, 76).

##### 3.2.2.3 EIF1AX

EIF1AX (located on chromosome 10) encodes for the X-linked Eukaryotic Translation Initiation Factor 1A protein (Eif1A), which regulates the initiation of protein translation. Its mutations lead to mis-selection of start sites, with suppressed translation of canonical transcripts or potential upregulation of oncogenes (77). Nevertheless, the exact biological function of EIF1AX and its contribution to tumorigenesis have not yet been fully clarified (70, 78).

EIF1AX mutations occur in 8% to 19% of all UM [58], in association with disomy-3 (60). Yavuzyigitoglu et al. showed that EIF1AX-mutated tumors have good prognosis and a low risk of metastasis (63, 79–81).

##### 3.2.2.4 SF3B1

SF3B1 (splicing factor 3b subunit 1), located on chromosome 2, encodes for a spliceosome’s component (82). Harbour et al. reported that 18.6% of UM mutations affect the SF3B1 gene (83).

Patients with SF3B1-mutated UM are younger at diagnosis (54.5 years of age) compared to patients with EIF1AX or BAP1 mutations (64 years) (84).

SF3B1-mutated UMs are mainly disomy 3 tumors with an intermediate level of metastatic risk, and metastases seem to occur later than in the presence of BAP1 mutations. Indeed, these patients have an apparent 34% risk of late-onset metastases (mean 11.2 years from diagnosis).

Exceptionally, SF3B1 or EIF1AX mutations occur in combination with monosomy 3, as well as BAP1 mutations in combination with disomy 3. Moreover, despite being defined as mutually exclusive, SF3B1 mutations can coexist with either EIF1AX or BAP1 mutations (85).

#### 3.2.3 Genome-Wide Expression Profiling

GEP classification has greater prognostic accuracy than cytogenetic methods, as it can be carried out on FNAB even when RNA quantity is below detectable limits, with a technical failure rate of only 3% (86). GEP analysis is also very sensitive for capturing overall tumor functional complexity in heterogeneous tumors with its simultaneous evaluation of several genes involved in the tumor microenvironment (87). Onken e al proposed a platform for predicting the risk of UM metastasis based on a 15-gene PCR-based assay (52, 88).

UMs can be classified based on GEP analysis as class 1 (low-risk), which are subsequently subdivided into 1A and 1B with 2% and 21% five-year metastatic risk, respectively, or class 2 (high-risk) tumors with a five-year metastatic risk of 72% (83).

The class 2 gene expression profile is strictly associated with classical factors of bad prognosis, such as larger tumor size, epithelioid cytology, extravascular looping matrix patterns, and monosomy 3 with BAP1 mutations. On the other hand, GEP class 1 tumors are characterized by disomy 3 and EIF1AX or SF3B1 mutations (89–91), but the prognostic accuracy of the class 2 expression profile is higher than many of these factors taken individually or in combination.

Preferentially expressed antigen in melanoma (PRAME) status has been recognized as an independent prognostic biomarker for UM, in fact it identifies increased metastatic risk in patients with Class 1 tumors. For this reason, GEP classification was revised based on PRAME status. It has been reported that when combined with a 12-gene expression panel, PRAME expression predicted a five-year metastatic rate of 0 in class 1/PRAME−, 38% in class 1/PRAME+, and 71% in class 2 tumors, respectively. PRAME expression is also positively correlated with larger tumor diameter and SF3B1 mutations as well as gain of 1q, 6p, 8q, and 9q and loss of 6q and 11q (92).

#### 3.2.4 Molecular and Biochemical Pathways Correlated With Metastasis Development

##### 3.2.4.1 Epithelial-to-Mesenchymal Transition

Epithelial-to-mesenchymal transition (EMT) represents an important event in the late stage of UM. With EMT, in relation to adherent-junction breakdown, cells acquire a spindle-shaped, highly motile fibroblastoid phenotype. Several transcription factors can regulate EMT, contributing to carcinogenesis and metastasis in many tumors with different histotypes, but little is known about UM. Early investigations demonstrated that ZEB1, Twist-related protein 1 (Twist1), and Snail Family Transcriptional Repressor 1 (Snail1) downregulation reduces the invasive properties of uveal melanoma cells, whereas elevated mRNA levels of ZEB1 and Twist1 are associated with a more aggressive clinical phenotype in uveal melanoma samples (93). Other authors have highlighted that EMT in UM cells can be initiated by long-term stimulation with proinflammatory cytokine IL-6. IL-6/STAT3 (Signal transducer and activator of transcription 3) signaling induces the transactivation of v-jun avian sarcoma virus 17 oncogene homolog (JunB), resulting in EMT changes. Therefore, the IL-6/STAT3/JunB pathway has a driving effect on the migration and invasion of UM cells (94).

##### 3.2.4.2 Cancer Stem Cells

Cancer stem cells are cells with self-renewal and multidirectional differentiation potential, leading to tumor invasion and metastasis (95). Recent reports indicate that the presence of cells with stem-cell-like features contributes to drug resistance, partially as a result of the EMT process (96, 97). Only a few studies have investigated the role of cancer stem cells in UM. Universally recognized markers of melanoma stem cells are lacking, while their pathways are far from being fully characterized (98). Though class 1 tumors show similarity to more mature neural crest cells and differentiated melanocytes, class 2 tumors are transcriptionally similar to primitive neural and ectodermal stem cells. Interestingly, class 2 tumors show no similarity to undifferentiated embryonic stem cells, suggesting that the class 2 signature does correspond to the emergence of a lineage-specific primitive transcriptional program, rather than a generalized ‘dedifferentiation’ (99).

##### 3.2.4.3 EMT-Associated Genes

As UM continues to genetically develop from primary tumor to metastatic disease, new genes other than those involved in pathogenesis has been discovered for the metastatic progression. Through GEP several authors have identified several genes involved in the EMT and linked to the presence of monosomy 3.

C-C chemokine ligands 18 (CCL18) gene was found to be the highest up-regulated. CCL18 has a chemotactic activity for naive T-cells, CD4+, and CD8+ T-cells, and plays a key role in creating of tumor-infiltrating lymphocytes (TILs). In contrast to other malignancies, the presence of tumor-infiltrating lymphocytes in UM has been associated with poor prognosis (100), but how specifically promote the disease progression has not been yet been established.

On the contrary, a high expression level of PTP4A3/PRL-3 (gene encoding protein tyrosine phosphatase type IV A member 3/protein of regenerating liver-3*)* is known to be highly predictive of metastasis by increasing UM cells migration *in vitro* and invasiveness *in vivo* (101–105). PTP4A3 acts directly and indirectly through the membrane accumulation of matrix metalloproteinase 14 (MMP14), a membrane-anchored metalloprotease with a central role in the extracellular matrix (ECM) remodeling, invasion and turnover needed for migration or invasiveness, representing a key metastatic event involved in oncogenesis (106, 107). Moreover, PTP4A3 correlated with the expression of several proteases like ADAM10 (A Disintegrin and Metalloproteinase 10), which is upregulated in melanoma metastases and related to many adhesion molecules that have a central role in developing malignant melanoma (108).

Using 2D phosphoprotein analysis, it has been discovered collapsing response mediator protein 2 (CRMP2) as a new target for PTP4A3. The presence of CRMP2 is a good prognostic factor, predictive of low risk for metastasis formation, as it slow down the UM cell migration and invasion process. PTP4A3 inhibit CRMP2 expression (109).

Motility and invasion is also promoted by other genes, like S100A4, a member of S100 family proteins, that have been implicated in tumor metastasis formation (110).

Another up-regulated gene is PRRX1 transcription factor, that facilitates the EMT, conferring migratory and invasive properties (111).

##### 3.2.4.4 Epigenetic Alterations

Epigenetics mechanisms have shown to be involved in UM. They include DNA methylation, chromatin remodeling, histone modification and non-coding RNAs (miRNAs).

###### 3.2.4.4.1 Methylation

Methylation in UM may involve tumor suppressor genes; among the others, RAS association domain family 1 isoform A (RASSF1A) and cyclin-dependent kinase inhibitor 2A (p161INK4a) have been deeply studied (112).

As RASSF1A is usually involved in cell-cycle regulation and apoptosis, a loss of function due to its promoter methylation has been associated with UM pathogenesis and metastatic progression (113).

Methylation of p161INK4 leads to a loss of function of the protein, with subsequent increased tumor cell proliferation and bad prognosis (114).

Hypermetilation of Ras and EF-hand domain containing (RASEF), in combination with homozygosity of RASEF, has been related with increased risk of death due to metastasis (115).

Recently, also BAP1 methylation has been recognized as an important prognostic marker of UM metastasis. Robertson et al. identified four different UM subtypes, based on chromosome 3 status and BAP1 methylation: two groups with bad prognosis and with monosomy 3 and two with better prognosis and with disomy 3. In the first group BAP1 showed a different DNA methylation if compared with that observed in the second group (116).

In recent years, several studies have highlighted that DNA methylation can be used to trace the tissue of origin of various tumors (117, 118). Jurmeister at al. investigated the possibility of using DNA methylation profiling to relate melanomas to their respective primary sites. They found out that only uveal melanomas are characterized by a different global DNA methylation profile, with distinct epigenetic signatures. Thus DNA methylation analysis differentiate uveal melanomas from melanomas of other primary sites (119).

Interestingly, it seems that the methylation patterns of primary tumors and metastases are different. Previous authors compared the methylation status of metastatic primary UM and their corresponding metastases and founded that in the latter case methylation events are likely random events or eventually patient specific (120).

###### 3.2.4.4.2 MicroRNAs

MicroRNAs (miRNAs or miRs) are a class of small (19 to 24 nucleotides) non-coding RNAs that are able to modulate target mRNA expression through sequence-specific interaction. Aberrant expression due to epigenetic modifications in miRNA has been linked to carcinogenic processes, altering many cellular activities, such as differentiation, proliferation, apoptosis and migration (24, 121, 122).

Despite numerous studies, our knowledge about miRNA–mRNA interactions remains poor. This is also because it is not a unique relationship: one miRNA can modulate several mRNA and one mRNA can bind to several miRNA (123, 124)

Recent researchers have found that altered expression levels of miRNA are one of the epigenetic mechanisms responsible for UM tumorigenesis (125). In fact, some miRNAs, which are highly accurate biomarkers of metastatic risk, have demonstrated the ability to affect cellular migration and invasions in UM (23, 126) (**Table 1**).

**Table 1:** miRNAs involved in UM metastasis and their target genes.

miRNA	Expression	Target gene	Mechanism
miR-454	↑	PTEN	↑ cell proliferation and invasion
miR-367	↑	PTEN	↑ cell proliferation and migration
miR-296-3p	↓	MMP-2, MMP-9	↓ cell proliferation and invasion
miR-20a	↑	Unknown	↑ cell proliferation, migration, and invasion
miR-155	↑	NDFIPI	↑ cell proliferation and invasion
miR-21	↑	P53	↑ cell proliferation and invasion
miR‐224‐5p	↓	PIK3R3/AKT3	↓ cell proliferation and invasion
MiR-23a	↓	Zeb1	↓ cell migration

Previous authors have highlighted how it is possible to split UM into two prognostic clusters based on miRNA expression. Specifically, 6 upregulated miRNAs are differently expressed as having low metastatic risk class 1 and high metastatic risk class 2 UM (127).

MiRNA can act as oncogenes or as tumor suppressors in UM. Phosphatase and tensin homolog (PTEN) is a tumor suppressor gene that can be regulated by several miRNAs, like MiR-454 and miR-367. An oncogenic role is played by regulating PTEN, which promotes UM cell proliferation, colony formation and invasion (128). MiR-296-3p targets matrix metalloproteinase (MMP) 2 and 9, which are closely related to UM angiogenesis and metastasis. Wang et al. (129) demonstrated that miR-296-3p transfection in UM cells could repress cell proliferation, migration and invasion by regulating MMP-2/MMP-9.

MiR-20a and miR-155 also have an oncogenic role in UM (130), by enhancing cell motility, proliferation and invasion.

MiR-21 is more highly expressed in UM cells than in uveal melanocytes (131). MiR-21 promotes cell migration and invasion by regulating p53 and its downstream targets glutathione S transferase pi (GST-Pi) and LIM and SH3 protein 1 (LASP1).

MiR‐224‐5p is down-expressed in UM cells (132). Its target genes are PIK3R3 (phosphatidylinositol 3 kinase) and AKT3 (protein kinase B). Therefore, miR‐224‐5p acts as a tumor suppressor *via* the miR‐224‐5p/PIK3R3/PI3K/AKT axis, which takes part in malignantly transforming various carcinomas. MiR-23a is involved in the epithelial-mesenchymal transition (EMT) process, which is important for initiating tumor metastasis. Previous research has shown that this begins with the loss of E-cadherin, a component in the adherent junction of epithelial cells (133). After loss of cell adhesion, epithelial cells can transform into mesenchymal cells and gain the ability to migrate and invade. Wang et al. (134) reported that miR-23a can degrade Zinc Finger E-Box Binding Homeobox 1 (ZEB1), by eliminating its suppression over E-cadherin, thus, in turn, miR-23a can upregulate E-cadherin, then reverse the EMT process in UM cells and finally decrease cellular migration capacity.

In order to deeply understand the effect of miRNA in the evolution of UM, Smit et al. studied the expression of their putative downstream mRNA-targets. They identified 4 target genes negatively correlated with miRNA expression and involved in the regulation of the cell cycle (HDAC4, CDK6, E2F8, and CCND2) (135).

Sharma et al. investigated the potential consequences of germline and somatic mutations in the miRNA binding region of the BAP1 gene. Overall, they found 69 target genes associated with these BAP1-associated miRNAs, which were distinct from other miRNAs associated with UM. Among the others, several chromatin-associated genes were founded to be target of these BAP1-associated miRNAs (136).

Van Essen showed a positive correlation between HLA Class I and the quantity if infiltration in UM by macrophages and lymphocytes. In addition, he found out that mRNA expression is associated with immune-histochemical staining for HLA Class I (137).

Therefore Souri et al. compared HLA expression with miRNA levels in the same tumors, using mRNA expression with four different HLA Class I probes. They identified two clusters of miRNAs, one that positively correlates with HLA Class I and infiltrating leukocytes, the other with an opposite relationship. Both miRNAs expression patterns in UM show a relation are to chromosome 3/BAP1 status. This study could be the starting point to consider the miRNAs as regulators of inflammation in UM, regulated by BAP1 (138).

## 4 Conclusions

Current learning on UM biology and genetics will enable further development of prognostic tests, as well as patient stratification based on long-term prognosis and metastatic risk. Moreover, advances in UM molecular characterization may support the development of therapeutic strategies by targeting relevant signaling pathways.

Despite its debated role, the genetic analysis could identify patients with high risk for metastasis, allowing a better management and follow-up of patients.

As also clinical, pathological, and radiological features have important role in determining UM prognosis, it would be valuable to use a staging system that incorporates both clinical and genetic data.

## 5 Dedication

We want to dedicate this review to our Master, Prof. Antonio Rossi (1924 – 2006), Head of University Eye Clinic and Rector Magnificus of Ferrara University, reporting the image of a cluster of metastasizing melanocytic cells within a new-formed tumor vessel, published in 1961 (Rossi A. and Alfieri G., Rass. Ital. Ottal., 30, 81,161, 1961, also published and quoted in Duke-Elder, S. Disease of the Uveal Tract, Vol.IX, in System of 401 Ophthalmology. Henry Kimpton Publishers, London, 1966 (**Figure 2**).

## Author Contributions

Conceptualization: CG, PT, MT, and PP. Validation: MT and PP. Writing—original draft preparation: CG, EF, GA, and SV. Writing—review and editing: CG, EF, and GA. Supervision: PT, MT, and PP. Project administration: PT. All authors have read and agreed to the published version of the manuscript.

## Conflict of Interest

The authors declare that the research was conducted in the absence of any commercial or financial relationships that could be construed as a potential conflict of interest.

## Publisher’s Note

All claims expressed in this article are solely those of the authors and do not necessarily represent those of their affiliated organizations, or those of the publisher, the editors and the reviewers. Any product that may be evaluated in this article, or claim that may be made by its manufacturer, is not guaranteed or endorsed by the publisher.
